# Comparative oncology: overcoming human cancer through companion animal studies

**DOI:** 10.1038/s12276-023-00977-3

**Published:** 2023-04-03

**Authors:** Ji Hoon Oh, Je-Yoel Cho

**Affiliations:** 1 Department of Biochemistry, Brain Korea 21 Project and Research Institute for Veterinary Science, Seoul National University College of Veterinary Medicine, Seoul, 08826 Republic of Korea; 2 Comparative Medicine Disease Research Center, Seoul National University, Seoul, 08826 Republic of Korea

**Keywords:** Tumour biomarkers, Cancer prevention

## Abstract

Comparative oncology is a field of study that has been recently adopted for studying cancer and developing cancer therapies. Companion animals such as dogs can be used to evaluate novel biomarkers or anticancer targets before clinical translation. Thus, the value of canine models is increasing, and numerous studies have been conducted to analyze similarities and differences between many types of spontaneously occurring cancers in canines and humans. A growing number of canine cancer models as well as research-grade reagents for these models are becoming available, leading to substantial growth in comparative oncology research spanning from basic science to clinical trials. In this review, we summarize comparative oncology studies that have been conducted on the molecular landscape of various canine cancers and highlight the importance of the integration of comparative biology into cancer research.

## Cancer: What dogs can teach us about human tumors

The study of naturally developing tumors in dogs can aid researchers in finding new treatments for all types of cancer, including cancer in humans. Ji Hoon Oh and Je-Yoel Cho from Seoul National University, South Korea, discuss how our canine companions, sharing our living conditions and with biological similarities to humans, provide an ideal comparative model for understanding the mechanisms of cancer and how to treat it. The authors review the overlap between canine and human cancers across a range of tumor types (including breast, lung and prostate) with an emphasis on genetic and epigenetic similarities that may offer new molecular targets for anticancer therapeutics. Once potential candidates are found, testing diagnostics and therapies on our four-legged best friends could help reduce the cost and time of drug development.

## An overview of cancer research

**Fig. 1:**
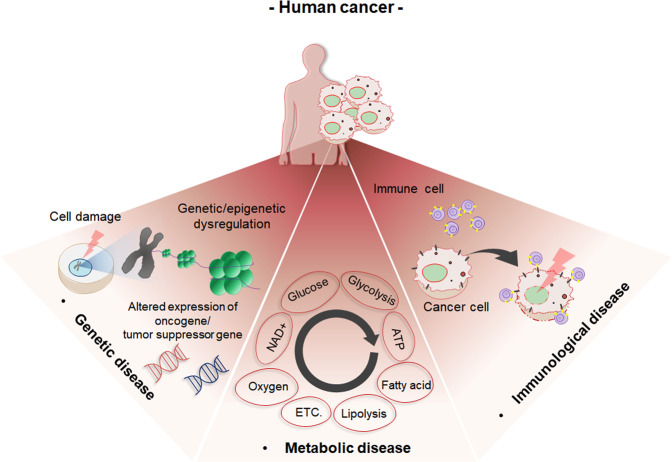
Schematic diagram outlining the cancer disease model. Cancer can be interpreted as a genetic disease, a metabolic disease, and an immune disease.

Cancer is a disease that affects populations worldwide, with one in three people developing cancer in their lifetime^1^. Cancer is usually characterized by the uncontrolled division of cells, which become malignant and form metastases that affect other healthy organs in the body^1^. Cancer can develop almost anywhere in the human body, which is made up of trillions of cells^2^. Normally, human cells grow and multiply to form new cells as the body needs them. When cells grow old or become damaged, they die, and new cells take their place^2^. Sometimes, however, when this orderly process is disrupted, abnormal or damaged cells grow and multiply when they should not. This can lead to the formation of tumors^2^. As tumorigenesis progresses, complex changes occur inside and outside the cell^1^. In particular, genetic mutations and epigenetic changes occur in cancer cells due to various factors^1,2^. Epigenetic changes, in particular, chromatin structure alterations due to DNA methylation and/or histone modification, occur and eventually lead to the dysregulation of oncogenes or tumor suppressor genes^1,2^. Tumorigenesis is also associated with cancer-related immune system problems^3^, including dysregulation of metabolism^4^. In addition, a cancer-specific tumor microenvironment forms extracellularly, leading to cancer spread and/or metastasis and enhancement of aggressive cell behaviors^5^. Various studies have focused on the genetic, metabolic, and immunological basis of cancer (Fig. 1). In addition to these studies, this review introduces comparative oncology research as a new perspective on cancer.

### Cancer as a genetic disease

Cancer has long been regarded as a genetic disease, and various studies have been conducted on its genetic basis^2,6^. These studies provide strong evidence for the genetic basis of cancer, i.e., the notion that cancer is a disease that results from the accumulation of genetic alterations, mutations, and epigenetic changes in key genes that regulate cell growth, division, and replication^2,6^. Depending on the effects of genetic alterations of key genes in cancer development, these genes are divided into oncogenes and tumor suppressors^2^. Genetic models of cancer development have provided important insights into the genetic processes that determine cancer initiation, progression, metastasis, the response to therapy, and the development of drug resistance^2,6^.

A representative example is that mutations in TP53 are found in ~38–50% of many cancers, including ovarian cancer, esophageal cancer, colon cancer, head and neck cancer, laryngeal cancer, and lung cancer cases, although they are not observed in all cancers^7^. The identification of targets based on the genetic characteristics of tumors, the development of tumor-specific drugs and the identification of patients who may benefit from such treatments are important challenges in overcoming cancer.

### Cancer as a metabolic disease

Recently, cancer has received much attention as a metabolic disease rather than a genetic disease. Several key metabolites identified in cancer (e.g., acetate, lactate, serine, sarcosine, asparagine, or choline) are found in almost all cancers, regardless of genetic modification^8^. The discovery of these different metabolic events may provide important insights into cancer and be useful for cancer diagnosis. For example, recent studies of metabolites in colon polyps and early-stage pancreatic cancer showed that metabolites can serve as biomarkers^9,10^.

Further evidence that cancer is a metabolic disorder is being confirmed by nucleocytoplasmic transfer research^11^. These studies aim to identify the origin of cancer by replacing damaged mitochondria or nuclei of cancer cells with normal mitochondria and nuclei^12^. If the cancer starts in a cell with a damaged nucleus, replacing it with a healthy nucleus should inhibit tumor growth. However, if the cancer originates from metabolic dysregulation due to mitochondrial dysfunction, restoring mitochondrial function may prevent cancer^12^. In this context, studies interpreting cancer as a metabolic disease are being conducted to overcome cancer, and these studies include targets such as glucose, glutamine, and fatty acid metabolism^11,12^.

### Cancer as an immunological disease

Much research has been conducted to interpret and treat cancer, both solid and hematological, as an immunological disease, and numerous approaches are being pursued worldwide. In this regard, it is very important to understand how the immune system influences the development and progression of cancer^3^. According to a recent study, immune escape due to tumor induction and tumor-induced alterations in the stromal tissue and immune system around the cancer mass is very important. Suppression of antigen exposure and presentation by malignant cancer cells, abnormal expression of certain chemokines and cytokines, induction of apoptosis in immune cells, and loss of immune cell function are associated with tumor evasion of recognition and elimination by the immune system^13^. Importantly, both adaptive and innate responses can be disrupted in the tumor microenvironment. As a result, many therapies modulating the immune system have been developed; these include immune checkpoint inhibitors and chimeric antigen receptor (CAR) T-cell therapies, which exploit a person’s immune system or immune cells to kill cancers.

Recently, there have been advances in methods of diagnosing and treating cancer in the new research area of molecular biology. Genetic, epigenetic, and omics approaches have provided a wealth of information to study the development and progression of cancer and to interpret different aspects of cancer, such as genetic, metabolic, and immunological aspects. Nevertheless, the mechanisms of tumorigenesis need to be further explored and investigated to find successful therapies for all types of cancer.

## Limitations in current cancer research

**Fig. 2:**
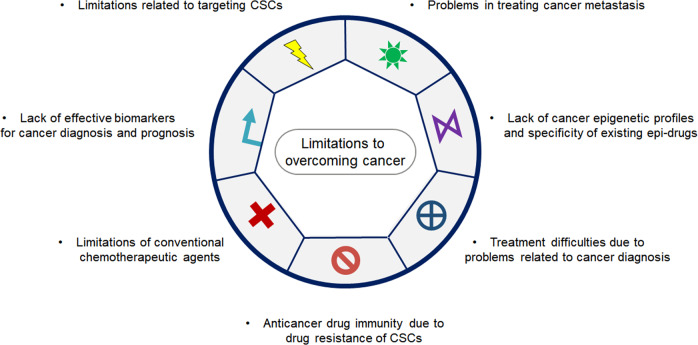
Limitations in current cancer research. There are still limitations to overcoming cancer for the following reasons: limitations regarding targeting of cancer stem cells (CSCs), anticancer drug immunity due to drug resistance of cancer stem cells, lack of cancer epigenetic profiles and specificity of existing epi-drugs, treatment difficulties due to problems related to cancer diagnosis, lack of effective biomarkers for cancer diagnosis and prognosis, limitations of conventional chemotherapeutic agents, and problems in treating cancer metastasis.

There are many proposed causes and mechanisms to explain the formation and progression of various cancers. Although there have been extensive studies on the pathogenesis of human cancer, there are numerous limitations associated with traditional preclinical research methods that tend to focus on cancer cells grown in 2-dimensional (2D) or 3D cultures or murine xenograft models to assess the efficacy of cancer agents; these limitations have contributed to the high drug attrition rates. In addition, there are some limitations to overcoming cancer^14^ (Fig. 2): (1) Limitations related to targeting cancer stem cells (CSCs), (2) anticancer drug immunity due to drug resistance of cancer stem cells, (3) lack of cancer epigenetic profiles and specificity of existing epi-drugs, (4) treatment difficulties due to problems related to cancer diagnosis, (5) lack of effective biomarkers for cancer diagnosis and prognosis, (6) limitations of conventional chemotherapeutic agents, and (7) problems in treating cancer metastasis^14^.

Therefore, the identification of novel biomarkers for human cancer and the discovery of new therapeutic candidates are essential to overcoming the major obstacles to improving existing therapies for the treatment and prevention of cancer. Ultimately, we highlight the importance of comparative research on cancer that occurs naturally in companion animals that share a living environment with humans as a new approach to studying cancer prevention and treatment.

## Comparative oncology: new insights into a human cancer

**Fig. 3:**
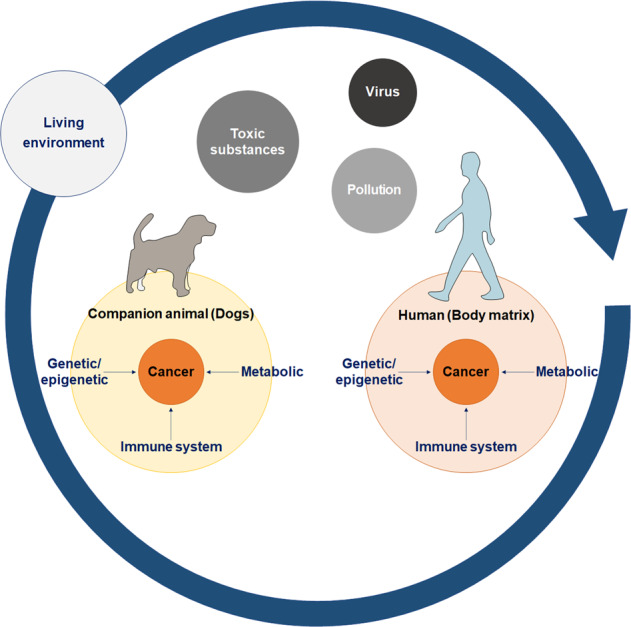
Schematic diagram outlining comparative medicine in companion animals and humans. Companion animals and humans share very similar living environments, exposing both species to similar toxic substances, viruses, and/or pollution. From these external stimuli, companion animals and humans develop cancer from genetic/epigenetic alterations, metabolic changes, and/or immune-related changes.

Comparative oncology is the study of cancers in companion animals for the determination of their translational relevance to human cancers^15^. Numerous types of cancers naturally occur in many types of companion animals, such as dogs, cats, rabbits, and horses^16,17^. A significant number of canine cancers are diagnosed every year, and dogs are very popular companion animals. Moreover, the ever-increasing accessibility of canine-specific reagents, resources, and scientific literature is opening up avenues for comparative oncology research between canines and humans (Fig. 3).

Much of the research on human cancers is based on mouse models due to their advantages, such as their small size and cost-efficiency^18^. Nevertheless, mouse models of cancer have limitations in mimicking human cancers because tumors arise spontaneously in humans, whereas tumor formation must be induced in mouse models. As a result, mouse models of human cancers usually lack any gene networks and interactions that account for tumorigenesis in humans.

Canines are excellent models for comparative oncology since they spontaneously develop the same types of cancers as humans. The histological types of these cancers are similar between dogs and humans. There is strong evidence that canines and humans share similar genes and pathways involved in tumorigenesis (Table 1). For example, it has been reported that BRCA1 and BRCA2 SNP markers are notably associated with mammary cancers in English Springer Spaniels and in breast cancers in humans^15^.

**Table 1:** Comparison of human and canine cancers.

Types of cancer	Alteration features	Alterations in human	Alterations in canine
Breast cancer	Signaling pathway	PI3K–AKT signaling	PI3K–AKT signaling
		WNT–β-catenin signaling	WNT–β-catenin signaling
		ERK signaling	ERK signaling
		p53 pathway -ATM, CHEK2, TP53, MDM2, and MDM4	p53 pathway -ATM, CHEK2, TP53, MDM2, and MDM4
	Molecular level (Mutation and/or loss/gain)	Mutations in PIK3CA	PIK3CA (A3140G) mutation
		ESR1, BRCA2	ESR1 and BRCA2
		CDKN2A, PTEN, CDH1, TP53, CSMD1, and PSD3	CDKN2A, PTEN, CDH1, TP53, CSMD1, and PSD3
		Mucin-1	Mucin-1
		BRCA1, IGF2R, FOXC2, DLG2 and USH2A	BRCA1, IGF2R, FOXC2, DLG2 and USH2A
		POLD1	POLD1
		MDM2, AKT3	
Prostate cancer	Molecular level (Mutation and/or loss/gain)	MDM2, PTEN, TP53, CTNNB1, CDH1, and ZBTB4	MDM2, PTEN, TP53, CTNNB1, CDH1, and ZBTB4
		AURKA	ATM, BRCA1, and MEN1
		NKX3.1 -Loss of PTEN	CDKN1B, NKX3.1, PTEN
			AR, TMPRSS2-ERG, TMPRSS2-ETV5
Lung cancer	Molecular level (Mutation and/or loss/gain)	EGFR and ALK	HER2^V659E^ mutation
		TP53, PTEN, SMAD4, KRAS, VHL, and HRAS	TP53, PTEN, SMAD4, KRAS, VHL, and HRAS
			Tyrosine kinase receptor (TKR)
Bladder cancer	Molecular level (Mutation and/or loss/gain)	BRAF^V600E^ mutation	Mutations in BRAF^V595E^, FAM133B, RAB3GAP2, and ANKRD52
		EGFR, HER2, CDKN2A, CDKN2B, PIK3CA, BRCA2, and NF-κB	EGFR, HER2, CDKN2A, CDKN2B, PIK3CA, BRCA2, and NF-κB
Glioma	Signaling pathway	RTK/RAS/PI3K signaling	RTK/RAS/PI3K signaling
		RB signaling	RB signaling
		p53 signaling	p53 signaling
	Molecular level (Mutation and/or loss/gain)	MGMT promoter methylation	CDKN2A and CDKN2B
		IDH1 or IDH2	PDGFRA
		chromosome 1p and 19q co-deletion	
Melanoma	Signaling pathway	PI3K–AKT signaling	PI3K–AKT signaling
		NF1, BRAF, and KIT	NF1, BRAF, and KIT
		PD-L1	PD-L1
			RAS family members
			TP53, PTEN, MYC, MDM2, and CDKN2A164–168
Lymphoma	Signaling pathway	NF-kB pathway	NF-kB pathway
	Molecular level (Mutation and/or loss/gain)		CD28, ABCA5, CCDC3 and SMOC2
Leukemia	Molecular level (Mutation and/or loss/gain)	Tyrosine kinase translocation	Tyrosine kinase translocation
		RB1	RB1
			c-KIT

In fact, 1 million out of 77 million dogs in the United States develop cancer each year; 50% of canine cancers develop in animals 10 or more years old, and a quarter of all dogs will develop cancer during their lifetime^19^. In this context, the United States launched the Canine Comparative Oncology Genomics Consortium (CCOGC) research project at the National Cancer Center (NCI) in 2004 to create a biorepository of canine cancer tissues and blood samples to decode their genes to conduct cancer research. Nevertheless, the last decade’s work on canine cancers focused on tumor biology, pathology, and genetics, and epigenetic pathways has not been thoroughly analyzed. Consequently, in this review, we aim to examine any epigenetic signatures that are shared by cancers in dogs and humans, which is of special interest. It is known that the noncoding regulatory regions of canine genomes are more similar to human genomes than mouse genomes are^20^. Our recent studies involving genomic and epigenomic comparisons across tissues of different species also revealed that chromatin map overlaps more between canines and humans (~40–50%) than between mice and humans (~10–20%). Furthermore, we also found that super enhancers were more highly conserved between canines and humans (~90% at ~50% minimum mismatch ratio) than between humans and mice (~30% at ~50% minimum mismatch ratio)^21^. Thus, the epigenetic changes affected by the environment might be more similar between canines and humans than between mice and humans. Therefore, we focused on comparing genetic and epigenetic aspects in canine and human cancers.

## Canine cancer models for comparative medicine

### Breast cancer

Human breast cancer became the most common cancer globally in 2021, accounting for 12% of new cancer cases worldwide according to the World Health Organization. Breast cancer is a disease in which malignant tumor cells form in the tissues of the breast, and it can be found in both men and women worldwide^22^. More specifically, breast cancer cells tend to form in the terminal ductal lobular unit, which is made up of the lobe and the ducts^23^. At the molecular level, breast cancer can be categorized into five molecular subtypes depending on the hormone receptors the cells express: Luminal A (ER/PR+, HER2−), luminal B (ER/PR+, HER2+), HER2-enriched (ER/PR−, HER2+), triple-negative (ER/PR−, HER2−), and normal-like (ER/PR+, HER2−, KI67−)^24^. These classifications are based on the presence or absence of estrogen, progesterone, and HER2 receptors. Breast cancer subtyping is important for treatment decision making^25^.

**Fig. 4:**
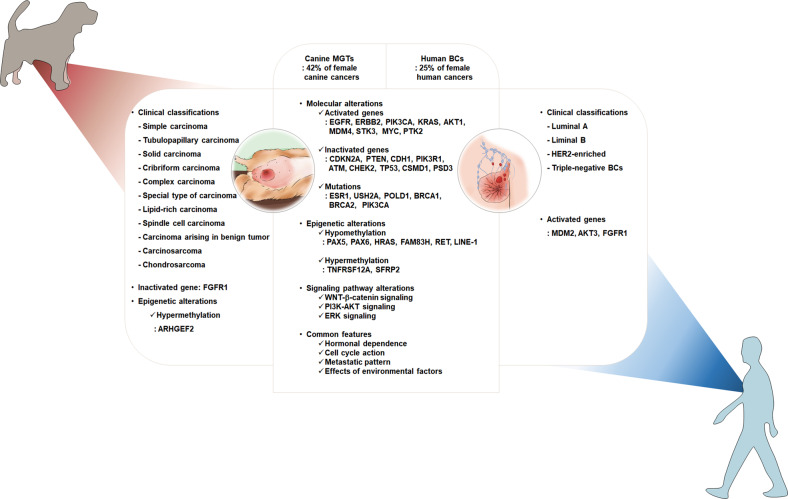
Comparison of human breast cancer and canine mammary gland tumor features. Canine mammary cancers (left panel) are typically categorized by histological subtype. On the other hand, human breast cancers (right panel) are usually categorized based on the presence or absence of hormone receptors. Numerous molecular and signaling pathway alterations (middle panel) are observed in both canine and human species.

Canine mammary tumors and human mammary tumors are similar in various aspects, such as hormonal dependence, metastasis pattern, relative age of onset, and role of environmental factors at the onset of the disease^26^. Approximately 60% of human cancers and 45% of canine breast cancers are estrogen receptor-positive^27^, and recent evidence suggests that many pathological and molecular similarities also exist between canine and human mammary tumors^28^. More recently, mammary tumor phenotypes found in humans, such as luminal A, luminal B and triple-negative (basal-like), have been identified in canines^29^. Comparative gene expression profiling and whole-exome sequencing studies between canine and human breast cancers revealed similarities such as cell cycle activation, WNT–β-Catenin signaling, PI3K–AKT and ERK signaling and mutations in ESR1 and BRCA2^30^. Moreover, loss of tumor suppressors such as CDKN2A, PTEN, CDH1 (which encodes E-cadherin) and TP53 was also observed in canine mammary tumors^30^ (Fig. 4). We previously performed high-throughput whole-exome sequencing using a total of 20 pairs of canine mammary gland tumors and adjacent normal tissues for genomic DNA isolation as a model for mammary gland tumors in dogs^31^. We found seven significantly mutated genes (SMGs) whose mutation rates were significantly higher than the background mutation rate in canine mammary tumors (CMTs) and reported that the *PIK3CA* gene was the most frequently mutated in CMT (45%). All somatic mutations identified in the *PIK3CA* gene resulted in corresponding amino acid sequence changes at six different loci, two variants (c.1637A > C and c.3140A > G) of which were identified as hotspots in CMT^31^. Analysis of an additional 62 CMT specimens reported that ~18 (~29%) harbored the *PIK3CA* (A3140G) mutation. The hotspot mutations in canine mammary gland tumors were an exact match for previously reported hotspot *PIK3CA* mutations in human breast cancer with a prevalence of ~30%^31^. This is very noteworthy from a comparative medicine point of view. Moreover, canine PIK3CA has a remarkable 99.8% DNA sequence identity to human *PIK3CA* and 99% amino acid identity to the encoded protein^31^. The location of the hotspot somatic mutation [nt 3140A > G (aa 1047His > Arg)] was exactly the same in both species^31^. In this respect, the mutational information of naturally occurring canine cancer provides valuable perspectives for translational comparative medicine studies for human cancer.

HER2 status has been investigated in numerous studies in canine mammary tumors to emphasize the similarity between canine and human cancers^32^. Multiple methods have been used to identify and classify HER2 expression in canine mammary tumor tissues; however, there is still a lack of validated methods for canine-specific HER2 detection, scoring, and clinical relevance^33^. More specifically, the association between HER2 status and tumor stage, grade, or clinical outcome still needs to be analyzed in canine mammary tumor models. Therefore, the status and role of HER2 expression in canine mammary tumors still needs to be further scrutinized to determine if it is a diagnostic, therapeutic and/or prognostic marker.

In an epigenetic study, our group first comprehensively profiled CMT methylation and inspected its correlation with the human breast cancer methylome^34^. We also suggested that changes in intron methylation play an important role in CMT by altering TF binding affinity^34^. The importance of intron methylation was further confirmed in human breast cancer data by the anti-correlation of LRIG1 gene expression with intronic hypermethylated PAX5 and hypomethylated PAX6 motifs^34^. Genome-wide methylation profiling was also performed in CMT and adjacent normal tissues coupled with matching PBMCs obtained from canines^35^. Methylation profiling in CMT identified miRNA candidates associated with human breast cancer. This study successfully revealed CMT-enriched differentially methylated regions (DMRs) in both tissues and PBMCs, and the putative roles of DMRs were characterized by GO and pathway analysis of associated genes^35^. As expected, many apoptosis-related genes, including ARHGEF2, TNFRSF12A, and SFRP2, were hypermethylated in CMT, and some oncogenes in human cancers, such as HRAS, FAM83H, and RET, were found to be hypomethylated^35^. Ultimately, these results suggest that molecular similarities between CMT and human breast cancer exist not only at the genomic and transcriptomic levels but also at the epigenomic level. As another example of epigenetic regulation, we assessed the diagnostic value of repeated, abundant, but strongly cancer-associated LINE-1 methylation in cfDNA isolated from small amounts of plasma from CMT and HBC subjects in previous studies^36^. Canine LINE-1 hypomethylation clearly differentiated subjects with CMT from healthy controls, and the same approach was applied to human breast cancer^36^. Altogether, these data suggest that the comparative approach using a canine model might aid in the rapid development of a new diagnostic biomarker and that the methylation of LINE-1 in cfDNA may be a good t a diagnostic marker for both human BC and CMT^36^.

Additionally, we sequenced total RNA from ten pairs of CMT tissues and matching adjacent normal tissues to identify CMT-associated transcriptomic signatures^37^. By comprehensive transcriptome analysis, 351 differentially expressed genes (DEGs) were identified in CMT^37^. Comparative analysis based on the DEGs revealed correlations between the three histological subtypes of CMT (ductal, simple, and complex) and four molecular subtypes of human BC (HER2+, ER+, ER&HER2+, and TNBC)^37^. Eight DEGs shared by all three subtypes of CMT had been previously reported as cancer-associated genes in human studies^37^. In addition, we previously published comparative medical studies with proteomics analysis in human breast cancer and CMT^38^. In the study, comparative analysis of canine and human cases revealed that the plasma protein LCAT was found a biomarker for advanced breast cancer as well as mammary tumors undergoing metastasis^38^.

Although limited, these reports indicate that canine mammary tumors share numerous downstream oncogenic alterations with human breast cancers and suggest potential for comparative research and drug development.

### Prostate cancer

Prostate cancer is the second most common cancer and the fifth leading cause of cancer-related death in men worldwide^39^. Prostate cancer is also found in canines, and it is more serious than in humans since prostate cancer is usually diagnosed at advanced stages in dogs, resulting in short overall survival and poor quality of life^40,41^. The incidence of prostate cancer in both species constitutes a model for therapies for advanced prostate cancers in humans^42^. Prostate cancer can be treated with local and systemic therapies and with nonsteroidal anti-inflammatory drugs (NSAIDs) in both canines and humans^43,44^.

Nevertheless, a large difference exists in that human prostate cancer is dependent on androgens, whereas canine prostate cancer is androgen-independent^45^. In the male reproductive system, androgens play a key role in the testes and adrenal glands, producing steroid hormones such as testosterone and dihydrotestosterone^46^. These hormones physically bind to androgen receptors, ultimately regulating gene expression that is involved in protein secretion, gene fusion, cell growth stimulation, growth factor production, and cell cycle regulation^47^. As a result, androgen receptors are directly responsible for the onset and progression of prostate cancers with numerous underlying mechanisms, such as receptor amplification or mutation, androgen biosynthesis changes, and/or androgen receptor binding cofactor changes, resulting in transcriptional activity modification^48,49^. However, there are now reports showing that many human prostate cancer patients develop the disease through pathways unrelated to androgen receptors^50,51^. In this line, canine prostate cancer, which is unaffected by androgen receptor aberrations, can serve as a good model. Not only does it mirror androgen-independent human prostate cancer, but it is also clinically similar to hormone-resistant human prostate cancer.

Consequently, comparative medical techniques can be utilized to characterize any DNA copy number aberrations, changes in signaling pathways, and expression of cancer-related genes, ultimately leading to alterations in molecular interaction networks. Moreover, canine-human interspecies cross-validation analysis revealed 79 genes that were simultaneously altered, further proving the molecular similarities behind human and canine prostate cancer^40^. These genes include ADRA1A, CCL17, CDH1, CFDP1, CHST4, CLU, CNGB1, CX3CL1, CYBA, EIF4A1, GALNS, GP1BA, GUCY2D, HSF4, MC1R, MX1, MYH1, NIP7, PLA2G15, SLC7A5, and TP53^40^. As an example, the tumor suppressor gene phosphatase and tensin homolog (PTEN) and oncogene signal transducer and activator of transcription 3 (STAT3) are known to be dysregulated in human prostate cancer and are linked to increased malignancy and a poor prognosis^52^. According to research, canine prostate carcinogenesis is also involved in the overexpression of STAT3 and downregulation of PTEN, and both indicators may be associated with the histological subtypes of prostate cancer and the degree of differentiation of neoplastic cells^52^. Moreover, VEGFR-2 appears to be an independent prognostic factor in animals with prostate cancers^53^. VEGF-A and VEGFR-2 are highly conserved between humans and canines^53^. In addition to the similarities, differences between canine and human prostate cancer exist. Highly prevalent alterations in human prostate cancer, such as gains of the MYC oncogene and deletions of the tumor suppressors NKX3-1, PTEN, RB1, and CDKN1B, were either absent or present in only very limited cases^40^.

### Lung cancer

Lung cancer is by far the leading cause of human cancer death, accounting for one-fourth of all cancer deaths^54^. Lung cancer can be classified largely into two histopathological subtypes: non-small-cell lung cancer (NSCLC; accounts for 85%) and small-cell lung cancer (SCLC; 15%). NSCLC can be further categorized into adenocarcinoma, squamous cell carcinoma, and bronchoalveolar and large cell carcinoma^54^. SCLC occurs in neuroendocrine cells of the bronchus^55^.

Unlike humans, lung cancer is rarely observed in dogs. The incidence is 1% in canines^56^. Surgery is the main form of therapy for both canines and humans; however, there is a heightened possibility of recurrence and metastasis in canines^57^. As a result, targeted systemic therapy, including conventional or immunotherapeutic reagents, is needed, and its development for canine use is crucial. The application of targeted systemic therapy for canines is receiving attention due to the success rate of immune checkpoint blockade therapy in humans^58,59^.

More importantly, even though canine lung cancers are infrequent, they can function as excellent comparative models for human NSCLC patients who have never smoked before. These human patients often have EGFR and ALK genetic mutations^60,61^. However, a few studies have shown that canine pulmonary adenocarcinomas do not show EGFR mutations or ALK alterations. Rather, in a study that analyzed 77 canine primary pulmonary carcinomas and 11 cell lines using whole-exome sequencing with selectively designed amplicons for 53 well-studied cancer genes, some commonly recurring mutated genes were included: HER2, TP53, PTEN, SMAD4, KRAS, VHL, and HRAS^32^. Therefore, although canine and human lung cancer models do share some clinical features, more studies need to be conducted to specify where there is biologic convergence and/or divergence to support molecular studies with targeted therapeutic agents in canine lung cancer patients for further validation in humans.

### Bladder cancer

Bladder cancer, also known as urothelial carcinoma (UC) or transitional cell carcinoma (TCC), is another type of cancer that is also found in both humans and canines^62,63^. Bladder cancer is a frequently occurring cancer in both men and women and can be categorized into nonmuscle-invasive bladder cancer (NMIBC) and muscle-invasive bladder cancer (MIBC), with NMIBC comprising ~80% of all bladder cancer patients^64^. NMIBC patients tend to have a good prognosis since this type of tumor is hardly invasive. Nevertheless, MIBC tumors tend to invade beyond the epithelial layer into the muscle^65^; therefore, the identification of their molecular signatures and molecular drivers is crucial.

Studies have revealed that the histological, biological, and clinical attributes are similar between human and canine bladder cancer^66–68^. Bladder cancer in both species shares molecular targets such as EGFR, HER2, CDKN2A, CDKN2B, PIK3CA, BRCA2, and NF-κB^16,69^. In particular, EGFR, which is overexpressed in more than 70% of human bladder cancer, is also observed in the canine patient population. Due to these molecular similarities, dogs are great models for the study of biomarkers and the development of therapeutic drugs for bladder cancer^68^. In addition, coordinated differential expression of genes within cytogenetic bands occurs in canine bladder cancer, and these patterns are similar to those found in human bladder cancer^66^. It was discovered that genes with mutations in canine bladder cancer are more likely than nonmutated genes to be downregulated at the transcriptional level in the tumor^66^. Moreover, the tumors tend to invade neighboring urinary tract structures or metastasize to loco-regional and remote sites in humans and canines^16^. Occasionally, similar but different molecular mutations arise. For instance, canine invasive urothelial carcinoma presents a BRAF^V595E^ mutation in 67–85% of cases, whereas human tumors harbor a BRAF^V600E^ mutation^70–72^. In this context, some new mutations (FAM133B, RAB3GAP2, and ANKRD52) were found for canine bladder cancer^66^. However, even though different mutations were identified in the two species, the fact that many molecular targets are shared between the two species of bladder cancer is an important aspect of comparative oncological study.

### Glioma

Intracranial gliomas are the most frequently occurring and one of the most lethal primary brain tumors in both humans and canines. In humans, gliomas are classified by progression, from low-grade (I–II) to high-grade (III–IV)^73^. Gliomas are one of the most frequently occurring brain tumors, especially in brachycephalic dog breeds^74,75^. Similar to humans, canines with gliomas display extremely poor survival despite various treatments ranging from chemotherapy radiation therapy to gene therapy^76–79^.

Studies on molecular alterations in GBM in humans have been investigated;^80–82^ the three main pathways involved are RTK/RAS/PI3K, RB, and p53 signaling^83^. Similar research has been conducted in canine gliomas, revealing genetic alterations in RTK/RAS/PI3K, RB, p53, CDKN2A, CDKN2B, and PDGFRA^84^. These genes and pathways are also found during human glioma genesis, revealing the similarities between human and canine glioma models^85^.

However, molecular phenotyping to differentiate human tumors based upon MGMT promoter methylation, mutation of IDH1 or IDH2 and chromosome 1p and 19q co-deletion has defined different prognostic subgroups, largely unrelated of histologic appearance, among human gliomas^86,87^. This is particularly relevant for human tumor samples that have a degree of mixed features and/or are complicated by insufficient and/or nonrepresentative sampling and provides avenues for targeted therapy development based upon molecular features.

### Melanoma

Melanoma is the most commonly occurring type of skin cancer in humans, usually due to exposure to the sun and ultimately UV rays^88,89^. Canine melanoma, on the other hand, usually does not occur on the outer skin, as it is sun-protected by their coat. Rather, canine melanoma frequently occurs within oral cavities and nail beds^90,91^. The treatment of melanoma is still difficult, as chemotherapy is not effective; however, the recent development of targeted therapy and immunotherapy has improved the prognosis of melanoma patients^92–94^. Melanoma is usually treated with surgical resection in canines; however, aggressive melanoma treatment cannot depend solely on surgery since the rate of metastasis is too high^90^. As a result, similar to humans, systemic chemotherapy drugs are needed to minimize metastasis^93^.

Human and canine melanoma share numerous similarities, making dogs a decent preclinical model to study melanoma^17,91^. Canine melanomas have mutations in the RAS family members TP53, PTEN, MYC, MDM2, and CDKN2A^95–97^. Interestingly, these genes have also been found to be altered in human melanomas. Furthermore, NF1, BRAF, and KIT oncogenic mutations have also been discovered in both species. ERK and/or PI3K signaling activation has also been identified in human and canine melanomas^98–100^. Moreover, PD-L1 expression has been detected in both canine melanoma cell lines and patient-derived tumor tissues, further elucidating the potential for the use of checkpoint inhibitors and/or immunotherapies to be applied to canines, as in humans^101,102^. Therefore, canine models can be used as representative models of human melanoma, especially in the development of next-generation therapies^103^. These reports suggest that canine melanoma may be particularly sensitive to checkpoint inhibitory antibodies or other immunotherapeutic modalities as they become available, which may reflect the success of such agents in melanoma therapy in humans^103,104^.

### Lymphoma

Lymphoma is a cancer of lymphocytes, which are immune cells that can usually be found in the lymph nodes, spleen, thymus, and bone marrow. Lymphoma can be categorized into two types: non-Hodgkin lymphoma (NHL) and Hodgkin lymphoma^105^. Lymphoma is found in both humans and canines, and multiple similarities exist, including cytogenetic and clinical features, pathology, tumor biology, tumor behavior, and genetic aberrations^106,107^. Consequently, canines can serve as an important animal model to study lymphoma and potential therapeutic options^108–110^.

One type of lymphoma is non-Hodgkin’s lymphoma (NHL), in which ~90% of cases are of B-cell origin in humans. On the other hand, the ratio of T-cell and B-cell lymphomas is 2:1 in canines, although there exists variance between breeds^111,112^. In a study involving a cohort of 608 canine lymphoma patients, 76% were found to have high-grade malignant lymphomas based on cytomorphological, histomorphological and immunological criteria and epidemiological and clinical data^111^.

Another type of lymphoma, diffuse large B-cell lymphoma (DLBCL), has been extensively studied in the canine model^113–115^. Gene expression profiling and immunohistochemistry analyses revealed that canine DLBCL has similar profiles to human DLBCL^113^. For example, NF-kB pathway genes are activated, and immunoglobulin heavy chain is altered^113^. Furthermore, germinal center and post-germinal center subtypes were identified in canine DLBCL, and these types showed different survival times; the findings in canines were consistent with DLBCL observations in humans^113^. In another study, gene expression profiles of 35 lymphoma samples in dogs were used to define three main groups: (1) low-grade T-cell lymphomas consisting exclusively of T-zone lymphomas; (2) high-grade T-cell lymphomas consisting of lymphoblastic T-cell lymphomas and peripheral T-cell lymphomas not otherwise specified; and (3) B-cell lymphomas consisting of marginal B-cell lymphomas, diffuse large B-cell lymphomas and Burkitt lymphomas^116^. The identified gene expression profiles were further categorized based on the expression of four genes related to lymphoma subtype and survival (CD28, ABCA5, CCDC3 and SMOC2)^116^. Moreover, a transcriptome comparison study based on RNA sequencing was performed with samples from 50 DLBCL patients and normal follicular B cells from 11 healthy dogs’ lymph nodes^117^. Transcripts involved in B-cell receptor (BCR), MYC signaling, the PI3K/AKT/mTOR pathway, DNA replication, and the cell cycle were significantly upregulated in DLBCL samples^117^. Furthermore, transcripts involved in the nuclear factor-κB (NF-κB) pathway (CD79, CD19, SYK, LYN, CARD11, BCL10, BTK, TRAF6, MYD88, NFKB2, TLR7, TLR9) were differentially expressed between DLBCL and normal samples^117^. Similar to these findings in canines, human DLBCL shows constitutive activation of NF-κB resulting from mutations in genes involved in this pathway^117^. These findings need further confirmation in larger cohorts of both humans and canines to evaluate the universal clinical utility of this comparative approach.

### Leukemia

Leukemia is a cancer of white blood cells that begins in the bone marrow. Leukemia is another hematologic malignancy that is equally common in dogs and humans. Numerous genomic studies in canine leukemia have been performed, revealing that the mechanisms behind leukemogenesis are similar between canines and humans^118^. For instance, in both species, RB1 is deleted in chronic lymphocytic leukemia (CLL), and BCR-ABL is fused in chronic myeloid leukemia (CML)^119^. In more detail, the BCR-ABL tyrosine kinase translocation, which is called the “Raleigh chromosome” in canines and the “Philadelphia chromosome” in humans, is being used for categorizing additional subtypes and is utilized in monitoring cytogenetic remission in CMLs^120–122^. Additionally, in acute lymphoblastic leukemia (ALL)/acute undifferentiated leukemia (AUL) and chronic lymphocytic leukemia (CLL), increased expression of c-KIT was observed^123^, suggesting the use of tyrosine kinase inhibitors as a treatment option for canine leukemia patients, and this treatment is commonly used in human leukemia patients with tyrosine kinase-related aberrations.

## Conclusion and perspectives

In this review, we explored several preclinical cancer models in both human and canine species that could be helpful for cancer research in terms of diagnosis, prognosis and treatment. Comparative medicine is a powerful tool and thus enables the development of novel therapeutic drugs. Currently, targeted therapies and personalized therapies are being actively developed, so the benefit of comparative medicine lies in that the selection of targets can be quickly and more easily made using animal targets. Through comparative medicine, researchers can identify new molecular targets, assess novel drugs, and identify which patient population would be fit for such novel therapies.

Numerous studies have been conducted to compare molecular profiles and tumor phenotypes in canine cancers and human cancers. Although further evaluation and clarification are necessary to associate canine cancers with human cancers, extensive studies have allowed the translation of diagnostic and prognostic markers to human oncology research. This review highlights the importance of canine models as ideal experimental models for studying and improving cancer treatments for humans.

## References

[1] Pruitt K (2016). Molecular and cellular changes during cancer progression resulting from genetic and epigenetic alterations. Prog. Mol. Biol. Transl. Sci..

[2] Hanahan D (2022). Hallmarks of cancer: new dimensions. Cancer Discov..

[3] Schreiber RD, Old LJ, Smyth MJ (2011). Cancer immunoediting: integrating immunity’s roles in cancer suppression and promotion. Science.

[4] Seyfried TN, Flores RE, Poff AM, D’Agostino DP (2014). Cancer as a metabolic disease: implications for novel therapeutics. Carcinogenesis.

[5] Neophytou CM, Panagi M, Stylianopoulos T, Papageorgis P (2021). The role of tumor microenvironment in cancer metastasis: molecular mechanisms and therapeutic opportunities. Cancers.

[6] Dancey JE, Bedard PL, Onetto N, Hudson TJ (2012). The genetic basis for cancer treatment decisions. Cell.

[7] Olivier M, Hollstein M, Hainaut P (2010). TP53 mutations in human cancers: origins, consequences, and clinical use. Cold Spring Harb. Perspect. Biol..

[8] Wishart DS (2015). Is cancer a genetic disease or a metabolic disease?. EBioMedicine.

[9] Harris H, Watkins JF, Campbell GL, Evans EP, Ford CE (1965). Mitosis in hybrid cells derived from mouse and man. Nature.

[10] Harris H, Watkins JF (1965). Hybrid cells derived from mouse and man: artificial heterokaryons of mammalian cells from different species. Nature.

[11] Seyfried TN, Shelton LM (2010). Cancer as a metabolic disease. Nutr. Metab..

[12] Seyfried TN (2015). Cancer as a mitochondrial metabolic disease. Front. Cell Dev. Biol..

[13] Shurin MR (2006). Intratumoral cytokines/chemokines/growth factors and tumor infiltrating dendritic cells: friends or enemies?. Cancer Metastasis Rev..

[14] Chakraborty S, Rahman T (2012). The difficulties in cancer treatment. Ecancermedicalscience.

[15] Schiffman JD, Breen M (2015). Comparative oncology: what dogs and other species can teach us about humans with cancer. Philos. Trans. R. Soc. Lond. B Biol. Sci..

[16] Dhawan D, Hahn NM, Ramos-Vara JA, Knapp DW (2018). Naturally-occurring canine invasive urothelial carcinoma harbors luminal and basal transcriptional subtypes found in human muscle invasive bladder cancer. PLoS Genet..

[17] Hernandez B (2018). Naturally occurring canine melanoma as a predictive comparative oncology model for human mucosal and other triple wild-type melanomas. Int. J. Mol. Sci..

[18] Onaciu A (2020). Spontaneous and induced animal models for cancer research. Diagnostics.

[19] Davis BW, Ostrander EA (2014). Domestic dogs and cancer research: a breed-based genomics approach. ILAR J..

[20] Paoloni M, Khanna C (2008). Translation of new cancer treatments from pet dogs to humans. Nat. Rev. Cancer.

[21] Son, K. H. et al. Integrative mapping of the dog epigenome: reference annotation for comparative inter-tissue and cross-species studies. Preprint at https://www.bioRxiv.org/content/10.1101/2022.07.22.501075v1 (2022). 10.1126/sciadv.ade3399 PMC10321747 37406108

[22] Feng Y (2018). Breast cancer development and progression: risk factors, cancer stem cells, signaling pathways, genomics, and molecular pathogenesis. Genes Dis..

[23] McCart Reed AE (2018). Mixed ductal-lobular carcinomas: evidence for progression from ductal to lobular morphology. J. Pathol..

[24] Lukasiewicz S (2021). Breast cancer-epidemiology, risk factors, classification, prognostic markers, and current treatment strategies—an updated review. Cancers.

[25] Waks AG, Winer EP (2019). Breast cancer treatment: a review. JAMA.

[26] Sultan F, Ganaie BA (2018). Comparative oncology: integrating human and veterinary medicine. Open Vet. J..

[27] Hansen K, Khanna C (2004). Spontaneous and genetically engineered animal models; use in preclinical cancer drug development. Eur. J. Cancer.

[28] Sorenmo KU (2009). Canine mammary gland tumours; a histological continuum from benign to malignant; clinical and histopathological evidence. Vet. Comp. Oncol..

[29] Varallo GR (2019). Prognostic phenotypic classification for canine mammary tumors. Oncol. Lett..

[30] Kim TM (2020). Cross-species oncogenic signatures of breast cancer in canine mammary tumors. Nat. Commun..

[31] Lee KH, Hwang HJ, Noh HJ, Shin TJ, Cho JY (2019). Somatic mutation of PIK3CA (H1047R) is a common driver mutation hotspot in canine mammary tumors as well as human breast cancers. Cancers.

[32] Lorch G (2019). Identification of recurrent activating HER2 mutations in primary canine pulmonary adenocarcinoma. Clin. Cancer Res..

[33] Seung BJ, Cho SH, Kim SH, Lim HY, Sur JH (2020). Quantitative analysis of HER2 mRNA expression by RNA in situ hybridization in canine mammary gland tumors: comparison with immunohistochemistry analysis. PLoS ONE.

[34] Nam AR (2020). Alternative methylation of intron motifs is associated with cancer-related gene expression in both canine mammary tumor and human breast cancer. Clin. Epigenetics.

[35] Jeong SJ, Lee KH, Nam AR, Cho JY (2019). Genome-wide methylation profiling in canine mammary tumor reveals miRNA candidates associated with human breast cancer. Cancers.

[36] Lee KH, Shin TJ, Kim WH, Cho JY (2019). Methylation of LINE-1 in cell-free DNA serves as a liquid biopsy biomarker for human breast cancers and dog mammary tumors. Sci. Rep..

[37] Lee KH, Park HM, Son KH, Shin TJ, Cho JY (2018). Transcriptome signatures of canine mammary gland tumors and its comparison to human breast cancers. Cancers.

[38] Park HM (2020). Common plasma protein marker LCAT in aggressive human breast cancer and canine mammary tumor. BMB Rep..

[39] Rawla P (2019). Epidemiology of prostate cancer. World J. Oncol..

[40] Laufer-Amorim R (2019). Comprehensive genomic profiling of androgen-receptor-negative canine prostate cancer. Int. J. Mol. Sci..

[41] Christensen BW (2018). Canine prostate disease. Vet. Clin. North Am. Small Anim. Pract..

[42] Leroy BE, Northrup N (2009). Prostate cancer in dogs: comparative and clinical aspects. Vet. J..

[43] Iizuka K (2022). Comparison of outcomes between medical and surgical treatment in dogs with prostatic adenocarcinoma: a retrospective study. BMC Vet. Res..

[44] Baek SJ, McEntee MF, Legendre AM (2009). Review paper: Cancer chemopreventive compounds and canine cancer. Vet. Pathol..

[45] Ryman-Tubb T (2022). Comparative pathology of dog and human prostate cancer. Vet. Med. Sci..

[46] Davey RA, Grossmann M (2016). Androgen receptor structure, function and biology: from bench to bedside. Clin. Biochem. Rev..

[47] Takayama K, Inoue S (2013). Transcriptional network of androgen receptor in prostate cancer progression. Int. J. Urol..

[48] Fujita K, Nonomura N (2019). Role of androgen receptor in prostate cancer: a review. World J. Mens. Health.

[49] Formaggio N, Rubin MA, Theurillat JP (2021). Loss and revival of androgen receptor signaling in advanced prostate cancer. Oncogene.

[50] Robinson D (2015). Integrative clinical genomics of advanced prostate cancer. Cell.

[51] Leung JK, Sadar MD (2017). Non-genomic actions of the androgen receptor in prostate cancer. Front. Endocrinol..

[52] Lin HY, Palmieri C (2016). Is STAT3 and PTEN expression altered in canine prostate cancer?. J. Comp. Pathol..

[53] Leis-Filho AF (2021). Expression and prognostic significance of vascular endothelial growth factor-A (VEGF-A) and its receptor in canine prostate cancer. Prostate.

[54] Nicholson AG (2022). The 2021 WHO Classification of lung tumors: impact of advances since 2015. J. Thorac. Oncol..

[55] Raso MG, Bota-Rabassedas N, Wistuba II (2021). Pathology and classification of SCLC. Cancers.

[56] Hifumi T, Miyoshi N, Kawaguchi H, Nomura K, Yasuda N (2010). Immunohistochemical detection of proteins associated with multidrug resistance to anti-cancer drugs in canine and feline primary pulmonary carcinoma. J. Vet. Med. Sci..

[57] Lee BM, Clarke D, Watson M, Laver T (2020). Retrospective evaluation of a modified human lung cancer stage classification in dogs with surgically excised primary pulmonary carcinomas. Vet. Comp. Oncol..

[58] Hellmann MD (2019). Nivolumab plus ipilimumab in advanced non-small-cell lung cancer. N. Engl. J. Med..

[59] Dafni U, Tsourti Z, Vervita K, Peters S (2019). Immune checkpoint inhibitors, alone or in combination with chemotherapy, as first-line treatment for advanced non-small cell lung cancer. A systematic review and network meta-analysis. Lung Cancer.

[60] D’Arcangelo M, D’Incecco A, Cappuzzo F (2013). Rare mutations in non-small-cell lung cancer. Future Oncol..

[61] Govindan R (2012). Genomic landscape of non-small cell lung cancer in smokers and never-smokers. Cell.

[62] Higuchi T (2013). Characterization and treatment of transitional cell carcinoma of the abdominal wall in dogs: 24 cases (1985-2010). J. Am. Vet. Med. Assoc..

[63] Park JC, Hahn NM (2014). Bladder cancer: a disease ripe for major advances. Clin. Adv. Hematol. Oncol..

[64] Zhu S, Yu W, Yang X, Wu C, Cheng F (2020). Traditional classification and novel subtyping systems for bladder cancer. Front. Oncol..

[65] Kobayashi T (2016). Understanding the biology of urothelial cancer metastasis. Asian J. Urol..

[66] Ramsey SA (2017). Cross-species analysis of the canine and human bladder cancer transcriptome and exome. Genes Chromosomes Cancer.

[67] de Brot S (2018). The dog as an animal model for bladder and urethral urothelial carcinoma: comparative epidemiology and histology. Oncol. Lett..

[68] Knapp DW (2014). Urinary bladder cancer in dogs, a naturally occurring model for cancer biology and drug development. ILAR J..

[69] Shapiro SG (2015). Canine urothelial carcinoma: genomically aberrant and comparatively relevant. Chromosome Res..

[70] Decker B (2015). Homologous mutation to human BRAF V600E is common in naturally occurring canine bladder cancer-evidence for a relevant model system and urine-based diagnostic test. Mol. Cancer Res..

[71] Mochizuki H, Breen M (2015). Comparative aspects of BRAF mutations in canine cancers. Vet. Sci..

[72] Mochizuki H, Kennedy K, Shapiro SG, Breen M (2015). BRAF mutations in canine cancers. PLoS ONE.

[73] Omuro A, DeAngelis LM (2013). Glioblastoma and other malignant gliomas: a clinical review. JAMA.

[74] Jose-Lopez R (2021). Clinical features, diagnosis, and survival analysis of dogs with glioma. J. Vet. Intern. Med..

[75] Merickel JL, Pluhar GE, Rendahl A, O’Sullivan MG (2021). Prognostic histopathologic features of canine glial tumors. Vet. Pathol..

[76] Pluhar GE (2010). Anti-tumor immune response correlates with neurological symptoms in a dog with spontaneous astrocytoma treated by gene and vaccine therapy. Vaccine.

[77] Boudreau CE (2021). Intratumoral delivery of STING agonist results in clinical responses in canine glioblastoma. Clin. Cancer Res..

[78] Miller AD, Miller CR, Rossmeisl JH (2019). Canine primary intracranial cancer: a clinicopathologic and comparative review of glioma, meningioma, and choroid plexus tumors. Front. Oncol..

[79] Hidalgo Crespo E, Farre Marine A, Pumarola IBM, Borrego Masso JF, Lujan Feliu-Pascual A (2022). Survival time after surgical debulking and temozolomide adjuvant chemotherapy in canine intracranial gliomas. Vet. Sci..

[80] Lai W, Li D, Kuang J, Deng L, Lu Q (2022). Integrated analysis of single-cell RNA-seq dataset and bulk RNA-seq dataset constructs a prognostic model for predicting survival in human glioblastoma. Brain Behav..

[81] Rajaraman P (2012). Genome-wide association study of glioma and meta-analysis. Hum. Genet..

[82] Couturier CP (2020). Single-cell RNA-seq reveals that glioblastoma recapitulates a normal neurodevelopmental hierarchy. Nat. Commun..

[83] Han S (2021). Alterations in the RTK/Ras/PI3K/AKT pathway serve as potential biomarkers for immunotherapy outcome of diffuse gliomas. Aging.

[84] Dickinson PJ (2016). Chromosomal aberrations in canine gliomas define candidate genes and common pathways in dogs and humans. J. Neuropathol. Exp. Neurol..

[85] Colardo M, Segatto M, Di Bartolomeo S (2021). Targeting RTK-PI3K-mTOR axis in gliomas: an update. Int. J. Mol. Sci..

[86] Kramar F (2016). IDH1/2 mutation and MGMT promoter methylation—the relevant survival predictors in Czech patients with brain gliomas. Folia Biol..

[87] Mizoguchi M (2012). Molecular characteristics of glioblastoma with 1p/19q co-deletion. Brain Tumor Pathol..

[88] Sample A, He YY (2018). Mechanisms and prevention of UV-induced melanoma. Photodermatol. Photoimmunol. Photomed..

[89] Sun X, Zhang N, Yin C, Zhu B, Li X (2020). Ultraviolet radiation and melanomagenesis: from mechanism to immunotherapy. Front. Oncol..

[90] Nishiya AT (2016). Comparative aspects of canine melanoma. Vet. Sci..

[91] Simpson RM (2014). Sporadic naturally occurring melanoma in dogs as a preclinical model for human melanoma. Pigment Cell Melanoma Res..

[92] Deacon DC, Smith EA, Judson-Torres RL (2021). Molecular biomarkers for melanoma screening, diagnosis and prognosis: current state and future prospects. Front. Med..

[93] Fonseca-Alves CE (2021). Current status of canine melanoma diagnosis and therapy: report from a colloquium on canine melanoma organized by ABROVET (Brazilian Association of Veterinary Oncology). Front. Vet. Sci..

[94] Shtivelman E (2014). Pathways and therapeutic targets in melanoma. Oncotarget.

[95] Hendricks WPD (2018). Somatic inactivating PTPRJ mutations and dysregulated pathways identified in canine malignant melanoma by integrated comparative genomic analysis. PLoS Genet..

[96] Prouteau A, Andre C (2019). Canine melanomas as models for human melanomas: clinical, histological, and genetic comparison. Genes.

[97] Wong K (2019). Cross-species genomic landscape comparison of human mucosal melanoma with canine oral and equine melanoma. Nat. Commun..

[98] Wei BR (2016). Synergistic targeted inhibition of MEK and dual PI3K/mTOR diminishes viability and inhibits tumor growth of canine melanoma underscoring its utility as a preclinical model for human mucosal melanoma. Pigment Cell Melanoma Res..

[99] Hayward NK (2017). Whole-genome landscapes of major melanoma subtypes. Nature.

[100] Fowles JS, Denton CL, Gustafson DL (2015). Comparative analysis of MAPK and PI3K/AKT pathway activation and inhibition in human and canine melanoma. Vet. Comp. Oncol..

[101] Hartley G (2017). Immune regulation of canine tumour and macrophage PD-L1 expression. Vet. Comp. Oncol..

[102] Maekawa N (2021). PD-L1 immunohistochemistry for canine cancers and clinical benefit of anti-PD-L1 antibody in dogs with pulmonary metastatic oral malignant melanoma. NPJ Precis. Oncol..

[103] Stevenson VB, Perry SN, Todd M, Huckle WR, LeRoith T (2021). PD-1, PD-L1, and PD-L2 gene expression and tumor infiltrating lymphocytes in canine melanoma. Vet. Pathol..

[104] Maekawa N (2022). Exploration of serum biomarkers in dogs with malignant melanoma receiving anti-PD-L1 therapy and potential of COX-2 inhibition for combination therapy. Sci. Rep..

[105] Jiang M, Bennani NN, Feldman AL (2017). Lymphoma classification update: T-cell lymphomas, Hodgkin lymphomas, and histiocytic/dendritic cell neoplasms. Expert Rev. Hematol..

[106] Seelig DM, Avery AC, Ehrhart EJ, Linden MA (2016). The comparative diagnostic features of canine and human lymphoma. Vet. Sci..

[107] Ito D, Frantz AM, Modiano JF (2014). Canine lymphoma as a comparative model for human non-Hodgkin lymphoma: recent progress and applications. Vet. Immunol. Immunopathol..

[108] Dias JNR (2021). Immunotherapeutic strategies for canine lymphoma: changing the odds against non-Hodgkin lymphoma. Front. Vet. Sci..

[109] McDonald JT (2018). Comparative oncology DNA sequencing of canine T cell lymphoma via human hotspot panel. Oncotarget.

[110] Marconato L, Gelain ME, Comazzi S (2013). The dog as a possible animal model for human non-Hodgkin lymphoma: a review. Hematol. Oncol..

[111] Ponce F (2010). A morphological study of 608 cases of canine malignant lymphoma in France with a focus on comparative similarities between canine and human lymphoma morphology. Vet. Pathol..

[112] Harris LJ (2020). Clinical features of canine nodal T-cell lymphomas classified as CD8+ or CD4-CD8- by flow cytometry. Vet. Comp. Oncol..

[113] Richards KL (2013). Gene profiling of canine B-cell lymphoma reveals germinal center and postgerminal center subtypes with different survival times, modeling human DLBCL. Cancer Res..

[114] Giannuzzi D (2019). Mutational landscape of canine B-cell lymphoma profiled at single nucleotide resolution by RNA-seq. PLoS ONE.

[115] Ferraresso S (2017). DNA methylation profiling reveals common signatures of tumorigenesis and defines epigenetic prognostic subtypes of canine diffuse large B-cell lymphoma. Sci. Rep..

[116] Frantz AM (2013). Molecular profiling reveals prognostically significant subtypes of canine lymphoma. Vet. Pathol..

[117] Aresu L (2019). New molecular and therapeutic insights into canine diffuse large B-cell lymphoma elucidates the role of the dog as a model for human disease. Haematologica.

[118] Avery AC (2020). The genetic and molecular basis for canine models of human leukemia and lymphoma. Front. Oncol..

[119] Breen M, Modiano JF (2008). Evolutionarily conserved cytogenetic changes in hematological malignancies of dogs and humans-man and his best friend share more than companionship. Chromosome Res..

[120] Culver S (2013). Molecular characterization of canine BCR-ABL-positive chronic myelomonocytic leukemia before and after chemotherapy. Vet. Clin. Pathol..

[121] Figueiredo JF, Culver S, Behling-Kelly E, Breen M, Friedrichs KR (2012). Acute myeloblastic leukemia with associated BCR-ABL translocation in a dog. Vet. Clin. Pathol..

[122] Perez ML (2013). Partial cytogenetic response with toceranib and prednisone treatment in a young dog with chronic monocytic leukemia. Anticancer Drugs.

[123] Giantin M (2013). Evaluation of tyrosine-kinase receptor c-KIT (c-KIT) mutations, mRNA and protein expression in canine leukemia: might c-KIT represent a therapeutic target?. Vet. Immunol. Immunopathol..

